# Difference in diel mating time contributes to assortative mating between host plant-associated populations of *Chilo suppressalis*

**DOI:** 10.1038/srep45265

**Published:** 2017-03-24

**Authors:** Wei-Li Quan, Wen Liu, Rui-Qi Zhou, Rong Chen, Wei-Hua Ma, Chao-Liang Lei, Xiao-Ping Wang

**Affiliations:** 1 Hubei Insect Resources Utilization and Sustainable Pest Management Key Laboratory, College of Plant Science and Technology, Huazhong Agricultural University, Wuhan 430070, P. R. China

## Abstract

Behavioral isolation in animals can be mediated by inherent mating preferences and assortative traits, such as divergence in the diel timing of mating activity. Although divergence in the diel mating time could, in principle, promote the reproductive isolation of sympatric, conspecific populations, there is currently no unequivocal evidence of this. We conducted different mate-choice experiments to investigate the contribution of differences in diel mating activity to the reproductive isolation of the rice and water-oat populations of *Chilo suppressalis*. The results show that inter-population difference in diel mating activity contributes to assortative mating in these populations. In the rice population, most mating activity occurred during the first half of the scotophase, whereas in the water-oat population virtually all mating activity was confined to the second half of the scotophase. However, when the photoperiod of individuals from the water-oat population was altered to more closely align their mating activity with that of the rice population, mate choice was random. We conclude that inter-population differences in diel mating time contribute to assortative mating, and thereby the partial reproductive isolation, of these host-associated populations of *C. suppressalis*.

Reproductive isolation in animals often evolves due to geographic barriers, habitat differences, or behavioral isolation, and can ultimately lead to speciation^1^,^2^,^3^,^4^,^5^,^6^,^7^. Assortative mating is a form of behavioral reproductive isolation that can reduce, or prevent, mating between individuals from different populations^8^,^9^,^10^. Assortative mating is defined as individuals preferentially choosing mates with a similar phenotype to themselves more often than would be expected under a null hypothesis of random mating^8^. Assortment traits are phenotypic traits such as mating sites^11^, the timing of mating activity^12^,^13^, body size^14^, body color^15^ and pheromones^16^, that influence the likelihood of mating between two individuals^17^. The mechanisms through which such traits contribute to assortative mating have been extensively documented in many animals^9^.

The timing of mating often has a diel rhythm, especially in insects^12^,^18^,^19^. Although it has been suggested that differences in diel mating time can potentially induce allochronic reproductive isolation between laboratory-bred strains of the same species^12^, closely related species^20^, and host-associated populations^21^,^22^, the evidence for this has so far been relatively equivocal. For example, although the rice and corn strains of *Spodoptera frugiperda* have different diel mating times, this is not the main reason for the reproductive isolation observed between these two strains in the wild^23^,^24^. There is still no evidence that divergence in diel mating time is a significant factor in the reproductive isolation of sympatric, conspecific populations.

*Chilo suppressalis* Walker (Lepidoptera: Crambidae), is an ideal species in which to investigate whether divergence in diel mating time contributes to reproductive isolation between host populations^22^,^25^. *C. suppressalis* is an economically significant pest of gramineous plants, especially rice and water-oats, in the East Asian region^22^,^26^,^27^. In this region rice and water-oats are usually cultivated in neighbouring fields, or planted in rotation in the same fields^28^,^29^ and are the only host plants on which *C. suppressalis* can complete its entire life-cycle without changing host^30^. Larvae, pupae, and adults, of *C. suppressalis* fed on rice plants, or collected from rice fields, are smaller than those fed on water-oat plants or collected from water-oat fields^25^,^26^,^28^,^31^,^32^. The seasonal peaks of emergence the rice population also differ from those of the water-oat population^26^,^28^,^29^. Furthermore, diel mating activity begins earlier in the rice population than in the water-oat population^22^,^33^,^34^,^35^. These differences suggest that the rice and water-oat populations of *C. suppressalis* are sympatric host-races^25^,^29^,^31^,^35^. Indeed, it has been suggested that differences in seasonal emergence and diel mating activity could be important factors contributing to the development and maintenance of reproductive isolation in these two host-associated populations of *C. suppressalis*^22^,^25^,^35^. However, because these populations have considerable overlap in the seasonal timing of adult emergence^29^,^35^, a slight overlap in the diel timing of mating activity^22^,^34^,^35^, and adults can migrate from rice to water oat fields, and vice versa^35^, there is potential for hybridization. Indeed, viable hybrid offspring have been produced under laboratory conditions^22^ and may exist in the wild^34^. Therefore, both the degree of assortative mating between these two host populations, and the importance of inter-population differences in diel mating time to this, remain unclear.

In this study, different mate choice experiments were designed with two different objectives. These were: (1) to confirm the degree of host-independent, behavioral, partial premating reproductive isolation (mating patterns) between the two host populations suggested by previous studies^22^,^25^,^35^, (2) to determine to what extent divergence in diel mating time contributes to the observed assortative mating of each population. To investigate the mating patterns of the two populations we conducted three kinds of mate choice tests; no choice, single choice (both female and male choice), and multiple choice, throughout the entire scotophase. To determine the contribution of inter-population differences in diel mating time to the mating patterns observed during the above experiments, we manipulated the photoperiod of individuals from the water-oat population to more closely align their peak period of mating activity with that of the rice population, and conducted mate choice experiments during the part of the scotophase in which there was the most overlap in mating activity between the two populations. To further clarify the contribution of inter-population differences in diel mating time to mating patterns, we confined some mate choice tests to the part of the scotophase that included the peak period of mating activity of the water-oat population, which was after the peak of mating activity of the rice population. The results show that these host-populations of *C. suppressalis* display a considerable degree of assortative mating and that inter-population differences in the diel timing of mating activity contributes to the premating isolation of these populations. This suggests that divergence in diel mating time can both maintain and reinforce the reproductive isolation of animal populations.

## Materials and Methods

### Insect populations

We collected > 1000 larvae of *C. suppressalis* from insect-damaged rice stems in a rice field (113°57′E, 30°29′N) and >800 from insect-damaged water-oat stems in a water-oat field (114°16′E, 30°28′N), in Wuhan, China in August 2014. Larvae were kept in an insectarium (temperature, 28 ± 1 °C; relative humidity, 80 ± 5%; photoperiod, light 15 h and dark 9 h). Rice (R) population larvae and water-oat (W) population larvae were reared on rice stems and water-oat fruit pulp, respectively. The time at which newly emerged adults of each population were first observed mating was recorded based on the methods of Samudra *et al*.^22^. A newly emerged male (1-day-old) and a newly emerged female (1-day-old) from the same population were randomly paired together and transferred into a clear plastic cup provided with 10% honey solution on a piece of cotton. The onset of mating was checked and recorded every 30 min throughout the scotophase. There was a significant difference in the diel peak mating activity of each population (Supplementary Fig. S1). This difference was considered to be an intrinsic feature of each host-associated population and to be independent of sampling time and locality^22^,^25^,^34^.

The diel peak of mating activity of the two host-populations of *C. suppressalis* is not affected by larval diet^25^. Nevertheless, an additional two complete generations of each population were reared on an artificial diet^36^ in the same insectarium under the conditions described previously between September and November 2014. The diel peaks of mating activity of the third generations of each population were still significantly different (Supplementary Fig. S1). Pupae from each population were sexed and sorted into four separate enclosures (R-males, R-females, W-males and W-females) and provided with 10% honey solution to prevent mating before experiments began. Newly emerged, 1-day-old adults of the third generation were used in mate choice experiments.

### Experimental design

**Figure 1:**
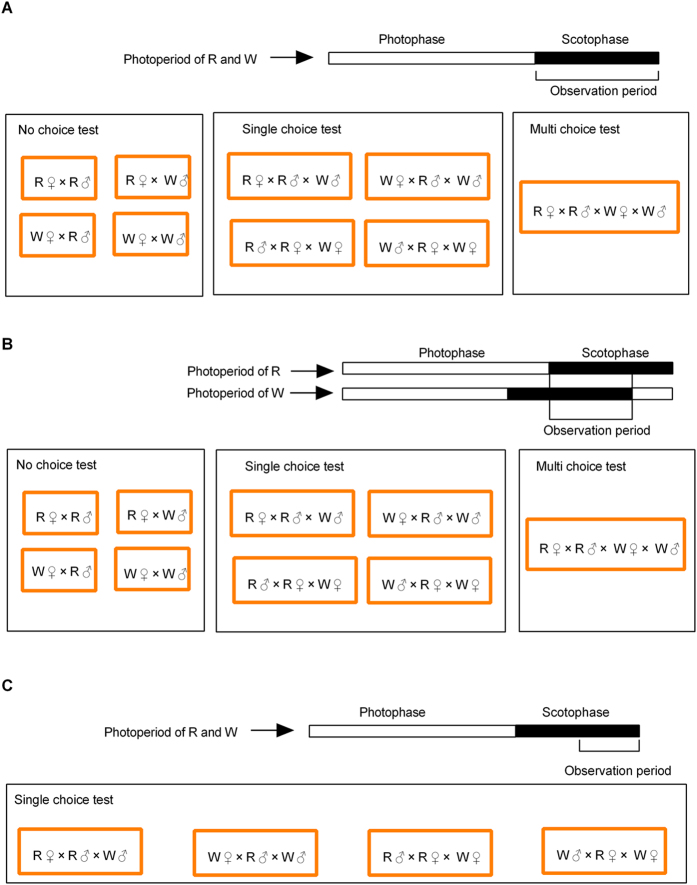
Schematic diagram of mate choice tests conducted on adults of the rice (R) and water-oat (W) populations of *C. suppressalis* during (**A**) the entire scotophase, (**B**) after the photoperiod of W individuals had been manipulated to align their peak of mating activity with that of R individuals, (**C**) during the peak period of mating activity of W individuals in the latter part of the scotophase. See Supplementary Table S1 and Materials and Methods for details.

The estimated degree of sexual isolation between sister species of *Drosophila* was significantly higher when experimental designs incorporated choice compared to those in which there was no choice^37^. Therefore, we used both choice, and no-choice, tests to determine the degree of premating, reproductive isolation (assortative mating) between the R and W populations of *C. suppressalis*. Tests were conducted by placing pairs, trios, or quartets, in a plastic cup before the beginning of the scotophase and observing their mating activity throughout the scotophase (Fig. 1A).

When the results of these tests indicated that mating was nonrandom, we conducted further experiments to determine whether inter-population differences in diel mating time contributed to the observed mating preferences of each population. Previous experiments had shown that virtually all mating activity in the W population was confined to the last 6–7 h of the scotophase. Although the peak of mating activity in the R population occurs in the first half (3–4 h) of the scotophase, some mating activity also occurs in the second half^25^. Therefore, to test the contribution of differences in diel mating time to intra-population mating patterns, we also conducted additional mate choice tests after synchronizing the mating activity of each population.

We did this in two ways. The first was by altering the photoperiod of some W adults so that their scotophase began 3 h earlier than those reared under the original photoperiod. We did this by rearing part of the third generation W population in another insectarium at the same temperature and humidity as the original insectarium, but, following the method described by Miyatake *et al*.^12^ and Schöfl *et al*.^23^, under a photoperiod in which the scotophase began 3 h earlier. Although the peak of mating activity in these photoperiod-altered W adults still took place during the last 6–7 h of the scotophase, there was greater overlap between their period of mating activity and that of R population adults than between ordinary W population adults and R population adults. Individuals were placed in the cups after the beginning of the scotophase and their mating activity observed during the final six hours of the scotophase (Fig. 1B).

The second method was, following Schöfl *et al*.^23^, to confine single choice tests to the latter part of the scotophase. We did this by placing chooser individuals into cups before the beginning of the scotophase, introducing their prospective mates 5 h into the scotophase and recording the start time of copulation throughout the remainder of the scotophase (Fig. 1C).

### Observation of the mating behavior in different choice tests

#### No choice tests

A virgin female and a virgin male were randomly paired together in a clear plastic cup and provided with 10% honey solution on a piece of cotton (Fig. 1A,B). Four different pair combinations (R♀ × R♂, W♀ × W♂, R♀ × W♂ and W♀ × R♂) were tested. Each pair was checked for copulation every 30 min over the duration of a single, given scotophase. The time at which copulation of each pair combination was first observed was recorded.

#### Single choice tests

A virgin female, or male, was placed in a clear plastic cup with two virgins of the opposite sex, one from the same, and one from the other, population, and provided with 10% honey solution on a piece of cotton (Fig. 1). Two female-choice experiments, R♀ × R♂ × W♂ and W♀ × R♂ × W♂, and two male-choice experiments, R♂ × W♀ × R♀ and W♂ × W♀ × R♀, were conducted. Males and females could be distinguished by their sexually dimorphic wing patterns. Individuals from each population were marked to distinguish them based on the methods described by Bailey *et al*.^38^. We did this by marking the tergum of individual moths with a black dot (Supplementary Fig. S2). To prevent bias, we alternately marked W and R individuals in successive choice tests. Marking did not significantly affect female (R population: χ^2^ = 0.02, *P* = 0.88; W population: χ^2^ = 0.07, *P* = 0.80) or male (R population: χ^2^ = 1.72, *P* = 0.19; W population: χ^2^ = 0.47, *P* = 0.49) mate choice. Copulations were scored at 30-min intervals. The population of copulating individuals, and the time at which copulation occurred, were recorded every 30 minutes. When multiple copulations occurred (5 cases in 420 choice trials), only the population and time of copulation of the first pair to copulate were recorded^38^.

#### Multiple choice tests

A virgin female and male from each population (R♀ × R♂ × W♀ × W♂) (Fig. 1A), were placed in a clear plastic cup, so that each had a choice of two potential mates. Individuals from each population were identified by marking as described above. The population, and sex, of the first pair to mate in each trial was recorded^38^.

All females and males used in experiments were 1 day old. All experiments were conducted over ten consecutive scotophases. About 180–320 choice trials were conducted in each scotophase depending on the numbers of adults that had emerged. Different choice experiments were randomized across the ten scotophases.

### Data analysis

The mating patterns and degree of sexual isolation between the W and R populations in different choice tests were estimated by the pair total index (PTI) and joint isolation index (I_PSI_)^37^,^39^. The PTI estimates whether the mating pattern of each pairing is different from random mating. PTI values significantly different from 1 indicate assortative mating between two populations. Mating patterns between two populations are considered assortative when I_PSI_ values are significantly different from 0. Although the contribution of differences in diel mating time is confounded by mate preference, I_PSI_ was also calculated for male-choice and female-choice tests to compare the degrees of sexual isolation estimated by different choice tests. A similar method of data analysis was used in previous studies^37^,^39^. The PTI and I_PSI_ were calculated using the JMATING software package^40^. The built-in statistical analysis program in this software estimated the significance of PTI and I_PSI_ by resampling 10,000 times and providing average PTI and I_PSI_ values^37^,^41^. Following Schöfl *et al*.^23^, we used a G–test to test whether the proportions of the four potential mating combinations were significantly different.

Female and males from each population may have contributed differently to the observed mating patterns. Therefore, following Korol *et al*. and Schöfl *et al*.^23^,^42^, a maximum-likelihood modeling approach was adopted to evaluate the respective contributions of divergence in the diel timing of mating activity and mate preference.

In brief, the model assumes that the probability of mating between a female and a male individual in single choice test is determined by (1) the female’s and male’s motivation to mate during the observation period, (2) the female’s or male’s degree of preference for individuals from same population versus those from another population. Eight parameters 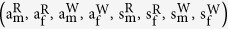 could be independently quantified by the number of intra- and inter-population copulations observed in single choice tests. The parameters 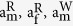 and 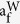 represent the mating activity of males and females of the R and W populations, respectively, and 
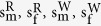
 represent the respective mating preference of males and females of these populations. The relative contribution of divergence in diel timing of mating activity and mating preferences can be effectively controlled and partitioned by the model. Some preconditions and assumptions are necessary for the model to be valid^23^,^42^ (for more details see Supplementary Methods).

Based on the numbers of each mating combination and unpaired individuals, we estimated a vector with the eight parameters by maximizing a likelihood function (see Supplementary Methods). This was accomplished by numerical optimization executed using the mle2 function in the R package bbmle. To evaluate the relative contributions of divergence in diel mating activity and mating preference, different constraints were used on the parameters (e.g. no difference in mating activity between the sexes of both populations, 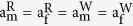). The likelihoods of models with different constraints could then be compared via likelihood ratio tests (LRTs)^23^,^42^ (See Supplementary Methods for details). This approach allowed us to infer the relative contributions of divergence in diel mating activity and mating preferences to the observed mating patterns. All analyses were performed in R-3.2.2 statistical software^43^.

## Results

### Results of mate choice tests conducted over the entire scotophase

**Figure 2:**
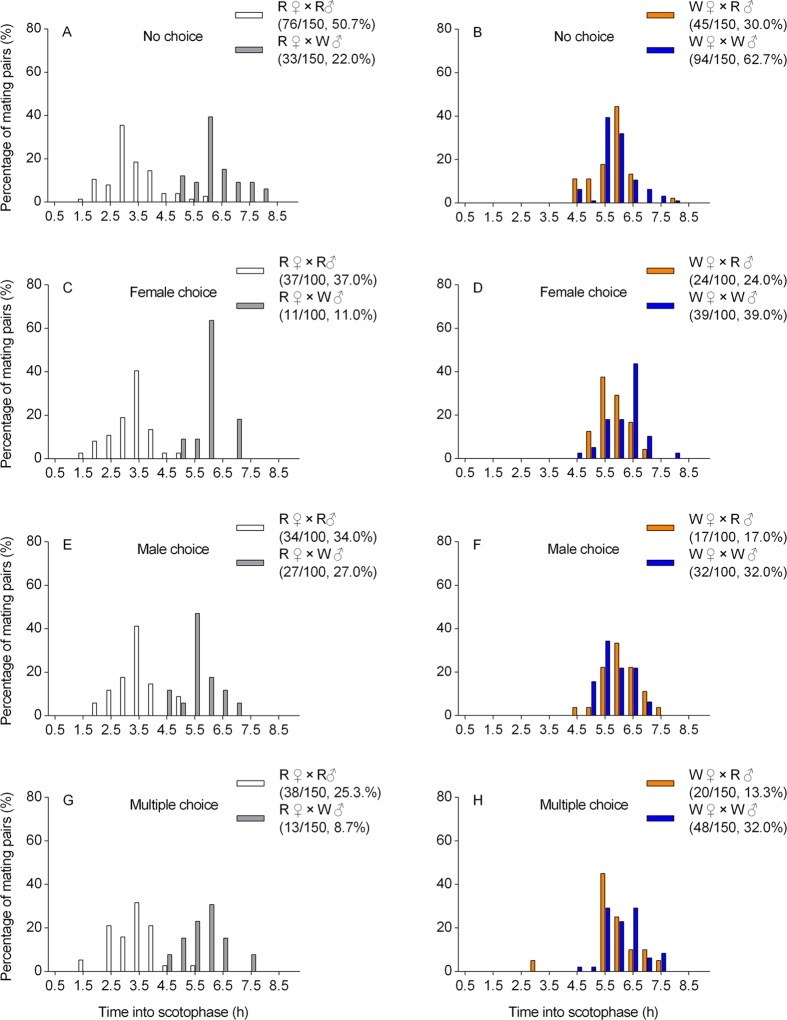
Temporal distribution of the beginning of the first copulation between males and females from the rice (R) and water-oat populations (W) of *Chilo suppressalis* in different mate choice experiments conducted over an entire scotophase. (**A**) no choice (R♀ × R♂ and R♀ × W♂); (**B**) no choice (W♀ × R♂ and W♀ × W♂); (**C**) female choice (R♀ × R♂ and R♀ × W♂); (**D**) female choice (W♀ × R♂ and W♀ × W♂); (**E**) male choice (R♀ × R♂ and R♀ × W♂); (**F**) male choice (W♀ × R♂ and W♀ × W♂); (**G**) multiple choice (R♀ × R♂ and R♀ × W♂); (**H**) multiple choice (W♀ × R♂ and W♀ × W♂). Sample sizes and proportion of different mating combinations shown in parentheses. The first value is the number of mating pairs and the second is the number of potential pairs; the percentage is the proportion of each specified pairing. See Supplementary Table S1 for details of sample sizes.

In no choice tests, the timing of the onset of copulation varied depending on whether the potential mate was from the same population or not (Fig. 2A,B). Copulation between R females and R males mostly occurred 3–4 h into the scotophase, but copulation between R females and W males mostly occurred 6–7 h into scotophase (Fig. 2A). Copulation involving W females mostly occurred 6–7 h into the scotophase, irrespective of the type of male present (Fig. 2B). The frequency of mating between homotypic pairs (R♀ × R♂, 76/150, 50.7%, W♀ × W♂, 94/150, 62.7%) was significantly higher than that between heterotypic pairs (R♀ × W♂, 33/150, 22.0%; W♀ × R♂, 45/150, 30.0%) (G-test of independence: G = 66.1, *P* < 0.001). PTI values were significantly > 1 for homotypic pairs but significantly < 1 for heterotypic pairs (Table 1). The overall I_PSI_ value was 0.38 ± 0.06 (*P* < 0.001). These results indicate that individuals preferred to mate with those from the same population even when they were not given a choice of mate.

In female-choice experiments (Fig. 2C,D), R females mated with R males (R♀ × R♂, 37/100, 37.0%) more frequently than with W males (R♀ × W♂, 11/100, 11.0%) (Supplementary Table S1). R females generally mated with R males in the first half of the scotophase, but generally mated with W males during the second half scotophase (Fig. 2C,D). W females also preferred to mate with W males (W♀ × W♂, 39/100, 39.0%), but did not reject R males (W♀ × R♂, 24/100, 24.0%) (Supplementary Table S1). The PTI value for R♀ × W♂ copulation was significantly < 1 (Table 2) whereas the PTI value for W♀ × W♂ copulation was significantly > 1 (Table 2) and overall I_PSI_ was 0.40 ± 0.09 (*P* < 0.001). This indicates that, when given a choice, females preferred to mate with a male from the same population.

In male-choice experiments (Fig. 2E,F), R males mated more often with R females (R♀ × R♂, 34/100, 34.0%) than with W females (W♀ × R♂, 17/100, 17.0%) (Supplementary Table S1), but W males mated almost as often with R females as with W females (W♀ × W♂, 32/100, 32.0%; R♀ × W♂, 27/100, 27.0%) (Supplementary Table S1). R males generally mated with R females (Fig. 2E) during the first half of the scotophase whereas copulation between all other potential combinations of mating pairs (W♂ × R♀, R♂ × W♀, W♂ × W♀) generally occurred later in the scotophase (Fig. 2E,F). The PTI value for W♂ × R♀ and W♂ × W♀ mating were neither significantly < 1 nor > 1 and the overall I_PSI_ value was 0.21 ± 0.09 (*P* = 0.02). These results indicate that, when given a choice of mate, males displayed a relatively weak preference for females from the same population.

In multiple choice experiments (Fig. 2G,H), copulation between homotypic pairs (R♀ × R♂, 38/150, 25.3% and W♀ × W♂, 48/150, 32%) was more common than between heterotypic pairs (R♀ × W♂ 13/150, 8.7% and W♀ × R♂ 20/150, 13.3%) (Supplementary Table S1). The peak of copulation between R females and R males occurred 3–4 h into the scotophase (Fig. 2G), whereas copulation between all other potential combinations of mating pairs (R♀ × W♂, W♀ × R♂, W♀ × W♂) took place 6–7 h into scotophase (Fig. 2G,H). PTI values for R♀ × W♂ and W♀ × R♂ mating pairs were significantly < 1, whereas the PTI value for W♀ × W♂ pairs was significantly > 1 (Table 2). The overall I_PSI_ value was 0.45 ± 0.08 (*P* < 0.001). These results indicate that assortative mating occurred when multiple males or females from each population were present in the same enclosure.

The onset of mating between R females and R males always occurred in the first half the scotophase, whereas almost all copulation involving W males or females took place in the second half of the scotophase (Fig. 2). These results suggest that these inter-population differences in diel mating time play an important role in assortative mating by R and W individuals.

### Results of mate choice tests conducted after synchronizing the mating activity of individuals from each population

To determine the degree to which the inter-population differences in diel mating activity contribute to the observed mating patterns of the two populations, mate choice experiments were repeated after the photoperiod of W individuals had manipulated to more closely synchronize their mating activity with those from the R population.

Under these conditions PTI values for all potential pairings in no choice experiments were not significantly different from 1 (Table 2) and the overall I_PSI_ was 0.03 ± 0.07 (*P* = 0.636), indicating no significant departure from the null hypothesis of random mating. I_PSI_ values obtained from single female and male choice experiments were 0.11 ± 0.11 (*P* = 0.311) and I_PSI_ = 0.07 ± 0.10, *P* = 0.486), respectively, which are also consistent with random mate choice. Mating was also random in multiple choice experiments (I_PSI_ = 0.07 ± 0.10, *P* = 0.499). These results suggest that inter-population differences in diel mating time are a major factor contributing to the assortative mating of the R and W populations of *C. suppressalis*.

### The relative contribution of individual mate preferences and inter-population differences in diel mating activity to assortative mating in each population

In order to estimate the relative contributions of the inter-population differences in diel mating activity and individual mating preferences to assortative mating in each population, we obtained different mating parameters from a maximum-likelihood model of single mate choice during different parts of the scotophase, and after synchronizing the mating activity of adults from both populations (Table 3).

When individuals were able to exercise mate choice throughout the scotophase, the mating activity between male and female individuals from the same population was not significantly different (Table 3: rice population, M_A1_ vs. M_A0_, *χ*^*2*^ = 0.00, *P* = 0.99; water-oat population M_A2_ vs. M_A1_, *χ*^*2*^ = 0.30, *P* = 0.59). The mating activity between individuals from the different populations was, however, significantly different (Table 3: M_A3_ vs. M_A2_, *χ*^*2*^ = 5.57, *P* = 0.02). Although females and males of the W population mated at random in single mate choice experiments (Table 3: male, M_A4_ vs. M_A2_, *χ*^*2*^ = 0.00, *P* = 0.98; female, M_A5_ vs. M_A4_, *χ*^*2*^ = 1.55, *P* = 0.21), males and females of the R population had a significant preference for mates from the same population (Table 3: male, M_A6_ vs. M_A5_, *χ*^*2*^ = 8.97, *P* = 0.003; female, M_A7_ vs. M_A5_, *χ*^*2*^ = 19.76, *P*** < **0.001).

When single choice tests were conducted after the photoperiod of W adults had been altered to synchronize their mating activity with that of R adults, the mating activity between individuals from the different populations was still significantly different (Table 3: M_B3_ vs. M_B2_, *χ*^*2*^ = 5.21, *P* = 0.02). However, females and males no longer had significant mating preferences (Table 3: M_B4_ vs. M_B2_, *χ*^*2*^ = 2.10, *P* = 0.14; M_B5_ vs. M_B4_, *χ*^*2*^ = 1.68, *P* = 0.19; M_B6_ vs. M_B5_ *χ*^*2*^ = 0.02, *P* = 0.90; M_B7_ vs. M_B6_, *χ*^*2*^ = 0.05, *P* = 0.86).

When mate choice was confined to the latter part of the scotophase, the mating activity within each population was not significantly different (Table 3: R population, M_C1_ vs. M_C0_, *χ*^*2*^ = 0.00, *P* = 0.99; W population M_C2_ vs. M_C1_, *χ*^*2*^ = 0.29, *P* = 0.59), but the mating activity between individuals from different populations was still significantly different (Table 3: M_C3_ vs. M_C2_, *χ*^*2*^ = 8.82, *P* = 0.003). R females and males now chose mates randomly (Table 3: male, M_C4_ vs. M_C2_, *χ*^*2*^ = 0.16, *P* = 0.90; female, M_C5_ vs. M_C4_, *χ*^*2*^ = 2.61, *P* = 0.11) but W males still significantly preferred females from the same population (Table 3: M_C6_ vs. M_C5_, *χ*^*2*^ = 6.10, *P* = 0.01). W females also preferred males from the same population, but this preference was not statistically significant (Table 3: M_C7_ vs. M_C5_, *χ*^*2*^ = 3.34, *P* = 0.06).

In summary, the diel timing of mating activity significantly differed between individuals from the R and W populations irrespective of which part of the scotophase choice experiments were conducted (Table 3). Individuals from R populations displayed significant mating preferences when mate choice experiments were conducted throughout the entire scotophase, but did not when experiments were confined to the latter part of the scotophase (Table 3). However, individuals from W populations displayed obvious mating preferences when experiments were confined to the latter part of the scotophase, but did not when experiments were conducted throughout the entire scotophase (Table 3). Individuals from both populations did not display significant mating preferences when the photoperiod of W adults had been altered to more closely synchronize their mating activity with that of R adults (Table 3).

## Discussion

Assortative mating is an important mechanism that establishes and maintains reproductive isolation between animal populations^4^,^8^. Assortative mating has two main components; mate choice and assortment traits^8^. Assortment traits are phenotypes which are expressed in both males and females that enhance the preference of individuals for mates with similar traits^8^. The importance of assortment traits, including mating site preferences, timing of seasonal emergence, and body size, in assortative mating has been well documented^11^. However, it remains unclear whether inter-population differences in diel mating time can produce a degree of assortative mating to contribute to the reproductive isolation of animal populations. The results of this study show that R and W populations of *C. suppressalis* display a significant degree of assortative mating, and that this is mainly due to inter-population differences in diel mating time.

The mating behavior of many insects has a diel rhythm which is controlled by an endogenous circadian clock^18^. In general, diel rhythms of mating activity are species-specific, and could play a key role in maintaining the reproductive isolation of closely related species^18^,^19^. For example, it has been suggested that allochronic variation in mating activity is a key factor in the reproductive isolation of sympatric strains of the fall armyworm *S. frugiperda*^21^,^44^. However, to date, the evidence for this has been equivocal. Indeed, behavioral experiments indicate that female mate preferences contribute more to the reproductive isolation of strains of this species than allochronic variation in mating activity^23^.

It has also been suggested that differences in diel mating activity could be an important factor maintaining the reproductive isolation of the R and W populations of *C. suppressalis*^22^,^25^,^35^. In the present study, I_PSI_ values ranged from 0.21 to 0.45, indicative of partial reproductive isolation, when individuals from each population were allowed to exercise mate choice throughout the entire scotophase. However, after the photoperiod of individuals from the W population was altered to more closely synchronize their mating activity with those of the R population, the estimated degree of reproductive isolation fell to almost zero. These results indicate that, in contrast to results obtained by Schöfl *et al*. on *S. frugiperda*^23^, inter-population differences in diel mating time make an important contribution to the reproductive isolation of these host-associated populations of *C. suppressalis*.

The results of this study show that different mate choice tests (e.g. female vs male choice) give different I_PSI_ values and similar variation has been reported in previous studies^23^,^37^,^39^. This is consistent with the hypothesis that the degree of reproductive isolation varies under different mating systems, and therefore, experimental designs^37^,^45^. Therefore, determining the degree of reproductive isolation between host-populations of *C. suppressalis* requires clarification of the mating system of this species. In moths in general, the mating process has two successive stages. In the first, female moths release sex pheromones that are used by males to locate them^46^. The initial mate choice is therefore made by males. Although it has been reported that the quantity of sex pheromones produced by females of the R and W populations differ^47^, field studies show that female sex pheromones of either population are equally effective at luring males of either population into traps^35^. For this reason we did not include a long-range, male-choice experiment in this study. In the second stage of the mating process, one, or more, males encounter a female, or females, which then choose a male to mate with^48^,^49^. During this stage, sex pheromones emitted by males may influence female choice and female sex pheromones may also continue to influence male choice. Thus, both males and females can potentially exercise mate choice during this, close proximity, stage of the mating process. In our study, the strength of male and female mate choice is likely to differ, which could explain the different I_PSI_ values obtained from our male choice (0.21), and female choice (0.40), experiments. The difference in these values is an estimation of the contribution of female, or male, mate choice to the partial reproductive isolation of these two populations. To explore the relative strengths of female and male choice in the second stage of the mating process, more detailed information on the courtship behavior of males and females is required, especially on the timing of pheromone emission^23^,^37^. Nonetheless, our results demonstrate that inter-population differences in the diel mating time of the R and W populations of *C. suppressalis* make a significant contribution to the partial reproductive isolation of these populations.

Traditional estimators of sexual isolation often confound inter-population differences in mating activity with individual mate choice^23^,^41^,^50^. For example, in the fall armyworm, there is a difference in the mating activity of the corn and rice strains of this species during the latter part of the scotophase, but not over the entire scotophase^23^. However, female preference to homotypic males in this species is due more to female mate choice than this difference in diel mating activity^23^. Although we didn’t observe courtship behavior in detail, which may have allowed us to separate the effects of inter-population differences in mating activity from mate choice^23^,^37^, the relative effect of these factors on mate choice can be effectively separated with a modeling approach^23^,^42^. We found differences in the mating activity of the R and W populations of *C. suppressalis* irrespective of which part of the scotophase was monitored. Moreover, the overall mating activity of the W population was higher than that of the R population during a specific part of the scotophase, which may explain the higher mating frequency of homotypic pairs of this population in no choice tests. We observed assortative mating in the R population when single choice tests were conducted throughout the entire scotophase, and assortative mating in the W population when tests were confined to the later part of the scotophase. However, there was no evidence of assortative mating by either population when mate choice tests were confined to the part of the later part of scotophase when the mating activity of both populations overlapped. These results suggest that the apparent preference to mate with individuals from the same population is actually due to inter-population differences in diel mating time. In other words, males or females will mate with any sexually active individual they encounter in the later portion the scotophase. This indicates an asymmetry of reproductive isolation between the two populations. Similar asymmetry in patterns of premating isolation has been reported in many insects^15^,^23^,^42^,^51^,^52^ and in fish^53^. In the R population, mating activity occurred throughout the entire scotophase but was more frequent in the first half of the scotophase, whereas in the W population almost all mating activity was confined to the second half of the scotophase. Copulation between heterotypic pairs was therefore almost always confined the second half of the scotophase, simply because individuals of the W population were rarely sexually active in the first half. Further research is required to investigate why the diel timing of mating activity is more variable in the rice population than in the water-oat population. In addition, because presence of leaves of water-oat plants promote mating by individuals from both populations^22^, it would be interesting to clarify whether the lower mating activity of the rice population is a fitness cost incurred by long-term adaption to rice.

In conclusion, our results suggest that inter-population differences in diel mating time make a significant contribution to the reproductive isolation of the R and W populations of *C suppressalis*. It should be noted, however, that viable hybrids are readily produced under laboratory conditions^22^ and evidence of asymmetric mating between the two populations was found in the present study. This suggests that the observed difference in diel mating time is unlikely to be the sole factor maintaining the partial reproductive isolation of these two populations in the wild. Indeed, the establishment and maintenance of the reproductive isolation of populations often requires the simultaneous, or successive, action of both pre- and post-mating isolation mechanisms^54^,^55^,^56^,^57^, for example, in some animals, body size can affect mating patterns^9^. Although high variation in body size within both populations has been reported in *C. suppressalis*^28^,^31^,^58^, there are also a significant inter-population differences in body size^25^,^26^,^28^,^31^,^32^. Therefore, to comprehensively evaluate the contribution of inter-population differences in diel mating time to the reproductive isolation of the R and W populations of *C. suppressalis* will require the investigation of other potential barriers to gene flow between these populations, such as size-assortative mating, seasonal allochronic isolation and habitat isolation. In addition, it would be interesting to investigate the ecological factors responsible for the divergence in diel mating times in these two populations. We speculate that diel mating time may be related to the timing of the release of host plant volatiles^59^. These are known to have a stimulatory effect on pheromone production and promote mating in insects^60^, but more work is needed to clarify their effect on *C. suppressalis*.

## Additional Information

**How to cite this article:** Quan, W.-L. *et al*. Difference in diel mating time contributes to assortative mating between host plant-associated populations of *Chilo suppressalis.* *Sci. Rep.* **7**, 45265; doi: 10.1038/srep45265 (2017).

**Publisher's note:** Springer Nature remains neutral with regard to jurisdictional claims in published maps and institutional affiliations.

## Supplementary Material

Supplementary Information

## Figures and Tables

**Table 1:** Estimates of degree of mate choice by males and females of the rice (R) and water-oat (W) populations of *Chilo suppressalis* in different mate choice tests conducted over an entire scotophase.

Mating situation	PTI	Value	SD	*P*
No choice	PTI_RR_	**1.23**	**0.12**	**0.043**
	PTI_RW_	**0.53**	**0.09**	**0.000**
	PTI_WR_	**0.73**	**0.10**	**0.009**
	PTI_WW_	**1.52**	**0.12**	**0.000**
Female choice	PTI_RR_	1.33	0.18	0.052
	PTI_RW_	**0.39**	**0.11**	**0.000**
	PTI_WR_	0.87	0.16	0.416
	PTI_WW_	**1.41**	**0.18**	**0.018**
Male choice	PTI_RR_	1.24	0.18	0.177
	PTI_RW_	0.98	0.16	0.891
	PTI_WR_	**0.62**	**0.14**	**0.008**
	PTI_WW_	1.16	0.17	0.348
Multiple choice	PTI_RR_	1.27	0.17	0.09
	PTI_RW_	**0.44**	**0.12**	**0.00**
	PTI_WR_	**0.67**	**0.14**	**0.03**
	PTI_WW_	**1.62**	**0.18**	**0.00**

Table show the PTI coefficients (estimates of mating preference) and their standard deviation (SD) for each mating pair combination. The JMATING software package was used to calculate the coefficients, their standard deviations, and significance (probability of rejecting the null hypothesis) by resampling the observed values 10,000 times (see Materials and Methods). RR represents a R♀ × R♂ mating, WW represents a W♀ × W♂ mating, RW represents a R♀ × W♂ mating and WR represents a W♀ × R♂ mating.

**Table 2:** Estimates of degree of mate choice by males and females of the rice (R) and water-oat (W) populations of *Chilo suppressalis* in mate choice tests after altering the photoperiod of one population to more closely synchronize its mating activity with that of the other.

Mating situation	PTI	Value	SD	P
No choice	PTI_RR_	0.91	0.12	0.436
	PTI_RW_	0.89	0.12	0.329
	PTI_WR_	1.04	0.13	0.810
	PTI_WW_	1.16	0.13	0.235
Female choice	PTI_RR_	1.02	0.18	0.920
	PTI_RW_	0.81	0.17	0.249
	PTI_WR_	0.98	0.18	0.876
	PTI_WW_	1.19	0.18	0.288
Male choice	PTI_RR_	0.95	0.17	0.723
	PTI_RW_	1.11	0.17	0.603
	PTI_WR_	0.76	0.15	0.111
	PTI_WW_	1.18	0.18	0.326
Multiple choice	PTI_RR_	0.95	0.17	0.694
	PTI_RW_	0.91	0.17	0.530
	PTI_WR_	0.95	0.17	0.706
	PTI_WW_	1.20	0.19	0.311

Table show PTI coefficients (estimates of mating preference) and their standard deviation (SD) for each mating pair combination. The software JMATING was used to calculate the coefficients, their standard deviations, and their significance (probability of rejecting the null hypothesis) by resampling the observed values 10,000 times (see Materials and Methods). RR represents a R♀ × R♂ mating, WW represents a W♀ × W♂ mating, RW represents a R♀ × W♂ mating and WR represents a W♀ × R♂ mating.

**Table 3:** Model parameters of the sexual behavior of *Chilo suppressalis* in single mate choice experiments.

Model	Rice population				Water-oat population				Likelihood	χ^2^ (df)	*H*_*0*_	*P*
	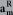	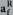	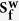	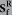	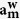	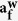	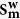	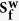				
A												
M_A0_: no restrictions	0.62	0.76	0.71	0.79	0.69	0.95	0.49	0.59	1429.10			
M_A1_: 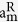 = 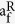	0.69	0.69	0.71	0.79	0.76	0.86	0.49	0.59	1429.10	0.00 (1)	M_A0_	0.99
M_A2_: 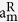 = 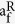 and 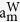 = 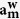	0.69	0.69	0.70	0.80	0.81	0.81	0.50	0.58	1428.95	0.30 (1)	M_A1_	0.58
M_A3_: 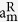 = 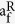 = 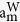 = 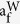	0.74	0.74	0.67	0.77	0.74	0.74	0.54	0.62	1426.17	5.57 (1)	M_A2_	**0.02**
M_A4_: M_A2_ and 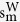 = 0.50	0.69	0.69	0.70	0.80	0.81	0.81	0.50	0.58	1428.95	0.00 (1)	M_A1_	0.98
M_A5_: M_A4_ and 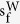 = 0.50	0.68	0.68	0.70	0.80	0.82	0.82	0.50	0.50	1428.18	1.55 (1)	M_A4_	0.21
M_A6_: M_A5_ and 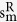 = 0.50	0.69	0.69	0.50	0.80	0.80	0.80	0.50	0.50	1423.69	8.97 (1)	M_A5_	**0.003**
M_A7_: M_A5_ and 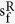 = 0.50	0.70	0.70	0.69	0.50	0.79	0.79	0.50	0.50	1418.29	19.76 (1)	M_A5_	<**0.001**
B												
M_B0_: no restrictions	0.68	0.59	0.58	0.64	0.94	0.66	0.49	0.46	1430.82			
M_B1_: 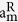 = 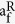	0.63	0.63	0.58	0.64	0.87	0.71	0.49	0.46	1430.82	0.00 (1)	M_B0_	0.99
M_B2_: 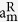 = 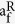 and 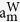 = 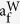	0.63	0.63	0.60	0.61	0.78	0.78	0.47	0.49	1430.33	0.98 (1)	M_B1_	0.32
M_B3_: 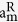 = 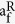 = 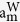 = 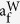	0.70	0.70	0.56	0.56	0.70	0.70	0.51	0.55	1427.73	5.21 (1)	M_B2_	**0.02**
M_B4_: M_B2_ and 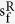 = 0.5	0.65	0.65	0.60	0.50	0.77	0.77	0.48	0.50	1429.28	2.10 (1)	M_B2_	0.14
M_B5_: M_B3_ and 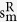 = 0.5	0.66	0.66	0.50	0.50	0.76	0.76	0.49	0.51	1428.44	1.68 (1)	M_B4_	0.19
M_B6_: M_B4_ and 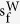 = 0.5	0.65	0.65	0.50	0.50	0.76	0.76	0.49	0.50	1428.43	0.02 (1)	M_B5_	0.90
M_B7_: M_B6_ and 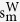 = 0.5	0.65	0.65	0.50	0.50	0.76	0.76	0.50	0.50	1428.42	0.05 (1)	M_B6_	0.86
C												
M_C0_: no restrictions	0.74	0.51	0.64	0.51	0.88	0.70	0.66	0.64	1443.44			
M_C1_: 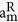 = 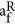	0.61	0.61	0.64	0.51	0.73	0.84	0.66	0.64	1443.44	0.00 (1)	M_C0_	0.99
M_C2_: 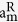 = 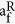 and 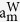 = 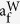	0.61	0.61	0.62	0.53	0.78	0.78	0.67	0.62	1443.29	0.29 (1)	M_C1_	0.59
M_C3_: 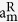 = 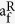 = 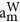 = 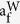	0.70	0.70	0.57	0.46	0.70	0.70	0.72	0.68	1438.88	8.82 (1)	M_C2_	**0.003**
M_C4_: M_C2_ and 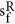 = 0.5	0.61	0.61	0.62	0.50	0.78	0.78	0.67	0.62	1443.21	0.16 (1)	M_C2_	0.90
M_C5_: M_C4_ and 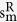 = 0.5	0.62	0.62	0.50	0.50	0.77	0.77	0.67	0.63	1441.91	2.61 (1)	M_C4_	0.11
M_C6_: M_C5_ and 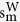 = 0.5	0.59	0.59	0.50	0.50	0.80	0.80	0.50	0.61	1438.86	6.10 (1)	M_C5_	**0.01**
M_C7_: M_C5_ and 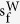 = 0.5	0.60	0.60	0.50	0.50	0.79	0.79	0.66	0.50	1440.23	3.34 (1)	M_C5_	0.06

A = single mate choice tests conducted throughout an entire scotophase, B = single mate choice tests conducted after the photoperiod of adults of the water-oat population had been altered to more closely synchronize their mating activity with those of the rice population, C = single mate choice tests confined to the second half of the scotophase including the peak period of mating activity of the water-oat population. The variables 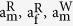,and 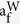 represent the mating activity of males (m) and females (f) of the rice (R) and water-oat (W) populations, respectively, and 
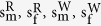
 represent the selectivity (mating preference) of males and females of these populations (see Materials and Methods). *P* values represent significant difference in the likelihoods of two models with different constraints, compared via likelihood ratio tests (see Materials and Methods).
