# Supplementary material for: Difference in diel mating time contributes to assortative mating between host plant-associated populations of Chilo suppressalis
Source: Sci Rep. 2017 Mar 24;7:45265. doi: 10.1038/srep45265 (PMC5364412; doi:10.1038/srep45265)
Supplement: Supplementary Information [file srep45265-s1.pdf]

Difference in diel mating time contributes to assortative mating between host plant-associated populations of *Chilo suppressalis*

Wei-Li Quan, Wen Liu, Rui-Qi Zhou, Rong Chen, Wei-Hua Ma, Chao-Liang Lei, Xiao-Ping Wang

Hubei Insect Resources Utilization and Sustainable Pest Management Key Laboratory, College of Plant Science and Technology, Huazhong Agricultural University, Wuhan 430070, P. R. China

Correspondence: xpwang@mail.hzau.edu.cn

Supplementary Information

Supplementary Methods

To estimate the separate contributions of divergence in the timing of mating activity and mate preference of both sexes from both populations in male and female mate choice tests. The maximum-likelihood modeling approach was used in a previous study for the same purpose[1,2].

The model assumes that a mating of female and male individual in single choice test is determined by (1) both the female's and male's mating drive during the observation period, (2) the female's or male's degree of preference for a mate from the same population. Eight parameters (a_m^R, a_f^R a_m^W, a_f^W s_m^R, s_f^R, s_m^W, s_f^W) could be independently quantified by the observed numbers of intra-population and inter-population copulations observed in a single choice test. The parameters a_m^R, a_f^R a_m^W, and a_f^W represent the mating activity of males and females in the rice and water-oat populations, respectively, and s_m^R, s_f^R, s_m^W, s_f^W represent the mating preferences of males and females of these population, respectively.

As shown in Supplementary Table 1, we could estimate the expected numbers of two pair combinations and one unpaired individual in single choice tests. The n is the total number of possible pairs (i.e., cups) for each choice test. For example, the frequency of W♀ × W♂ pairings could be calculated by multiplying the probability of a W female mating, the probability of a W male mating, and the probability of a W female choosing a W male in the W female choice test (Supplementary Table S1). The frequency of inter-population mating and unpaired individuals could also be calculated according to the related parameters. In fact, all the eight parameters were estimated by integrating the three possible outcomes in the four choice scenarios (Supplementary Table S2). Therefore, the relative contributions of

divergence in the diel timing of mating activity and mating preference could be effectively controlled and partitioned in the model.

A precondition for the model was that the mating preference of the choosing sex was modeled in single choice tests. However, the mating activity of all three individuals could be modeled using the relevant independent parameters. Moreover, the model hypothesizes that the mating activity of individuals in each trio was independent and that the mating preferences of females in female choice tests were not affected by the mating preference of males, and vice versa. In addition, the mating activity of females or males was supposed to be constant between female choice tests and male choice test.

We can estimate a vector θ = {a_m^R, a_f^R, s_m^R, s_f^R, a_m^W, a_f^W, s_m^W, s_f^W} by maximizing likelihood function as follows[2].

$$\ln L(\theta) = \sum_{i=1}^{3}\sum_{j=1}^{4} N_{ij} \ln n_{ij}(\theta)$$

The N_{ij} (i =1, 2, 3; j =1, 2, 3, 4) and $n_{ij}(\theta)$ are the observed numbers and expected numbers of each combination and unpaired individuals in four choice tests, respectively. This can be accomplished by numerical optimization which was executed in the mle2 function of the R package bbmle. To evaluate the relative contributions of divergence in the diel timing of mating activity and mating preference, different constraints were used on the parameters (for example: no difference in mating activity between both populations and sexes, $a_m^R = a_f^R = a_m^W = a_f^W$). The relative likelihoods of models (θ_1, θ_2) with different constraints could be compared via using likelihood ratio tests (LRTs) as follows[2].

$$G = 2[\ln L(\theta_1) - \ln L(\theta_2)]$$

The distribution of the G statistic approximates that of the chi-square distribution. Based on the results, we could infer the relative contribution of divergence in the diel timing of mating activity and mating preference to the observed mating patterns. All analyses were performed in R-3.2.2 statistical software[3]

References

1 Korol, A. *et al.* Nonrandom mating in *Drosophila melanogaster* laboratory populations derived from closely adjacent ecologically contrasting slopes at “Evolution Canyon”. *P. Natl. acad. sci. USA* **97**, 12637-12642 (2000).

2 Schöfl, G., Dill, A., Heckel, D. G. & Groot, A. T. Allochronic separation versus mate choice: nonrandom patterns of mating between fall armyworm host strains. *Am. Nat.* **177**, 470-485 (2011).

3 R Development Core Team. R: A Language and Environment for Statistical Computing, http://www.r-project.org/ (2015).

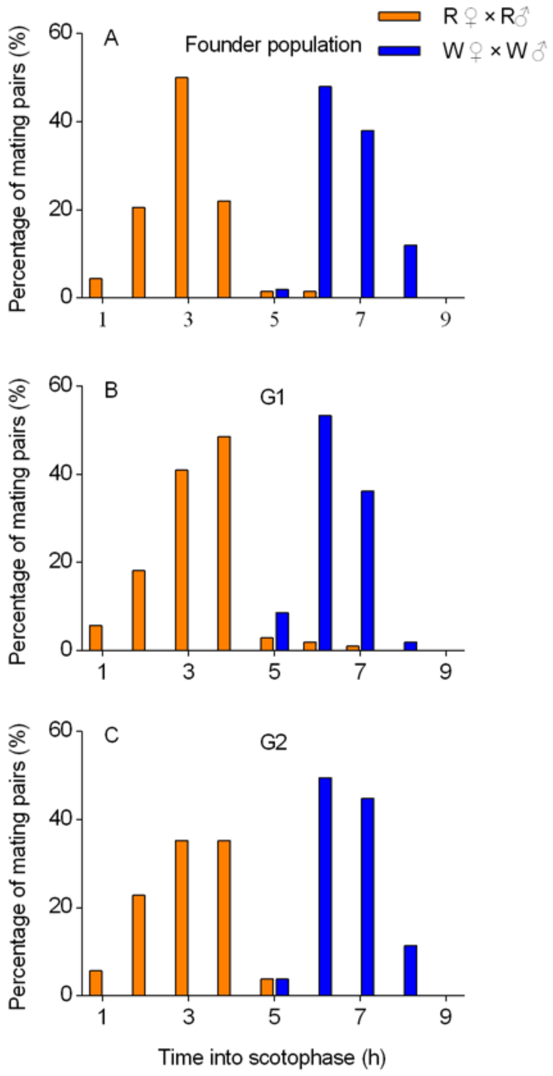

Supplementary Figure S1. Timing of the peak of mating activity (first copulation) of adults of the rice (R) and water-oat (W) populations of C*hilo suppressalis* collected as larvae from the wild, and of their descendants which were reared on an artificial diet. Numbers of W and R individuals were, respectively, 50 and 68 for the founder populations, 105 and 125 for the G1 populations and 108 and 115 for the G2 populations.

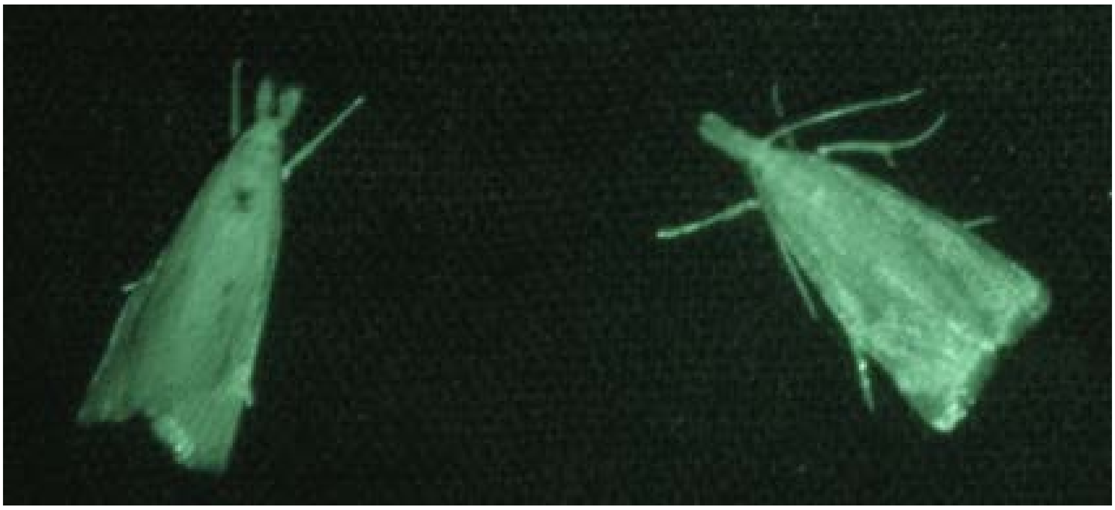

Supplementary Figure S2. Black dot marked on the tergum of a male *Chilo suppressalis*.

Supplementary Table S1 Numbers of pairs that mated and total number of potential pairs of adults from the rice and water-oat populations of *Chilo suppressalis* observed in mate choice experiments. (A) during the entire scotophase, (B) after the photoperiod of W individuals had been manipulated to align their peak of mating activity with that of R individuals, (C) during the peak period of mating activity of W individuals in the latter part of the scotophase. See Materials and Methods for details.

	A		B		C	
Mating situation	Mated pairs	Potential pairs	Mated pairs	Potential pairs	Mated pairs	Potential pairs
No choice						
R♀ × R♂	76	150	44	100	-	-
R♀ × W♂	33	150	43	100	-	-
W♀ × R♂	45	150	50	100	-	-
W♀ × W♂	94	150	56	100	-	-
Female choice						
R♀ × R♂	37	100	24	100	18	100
R♀ × W♂	11	100	19	100	21	100
W♀ × R♂	24	100	23	100	18	100
W♀ × W♂	39	100	28	100	38	100
Male choice						
R♀ × R ♂	34	100	25	100	25	100
W♀ × R ♂	17	100	20	100	19	100
R♀ ×W ♂	27	100	29	100	16	100
W♀ × W ♂	32	100	31	100	41	100
Multiple choice						
R♀ × R♂	38	150	23	150	-	-
R♀ × W♂	13	150	22	150	-	-
W♀ × R♂	20	150	23	150	-	-
W♀ × W♂	48	150	29	150	-	-

Supplementary Table S2 Expected numbers of intra-population pairs, inter-population pairs and unpaired individuals in single choice tests. The parameters a_m^R, a_f^R a_m^W, and a_f^W represent the mating activity of males and females of the rice and water-oat populations, respectively, and s_m^R, s_f^R, s_m^W, s_f^W represent the mating preference of males and females of these populations, respectively. The n is the total number of possible pairs (i.e., cups) for each choice test.

	Intra-population pair	Inter-population pair	Unpaired
Male-choice test			
**R**♂+R♀+W♀	$na_m^R a_f^R s_m^R$ （R♀+**R**♂）	$na_m^R a_f^R (1-s_m^R)$ （W♀+**R**♂）	$n[1-a_m^R a_f^R s_m^R - a_m^R a_f^W (1-s_m^R)]$
**W**♂+R♀+W♀	$na_m^W a_f^W s_m^W$ （W♀+**W**♂）	$na_m^W a_f^R (1-s_m^W)$ （R♀+**W**♂）	$n[1-a_m^W a_f^R (1-s_m^W)-a_m^W a_f^W s_m^W]$
Female-choice test			
**R**♀+R♂+W♂	$na_m^R a_f^R s_f^R$ （**R**♀+R♂）	$na_m^W a_f^R (1-s_f^R)$ （**R**♀+W♂）	$n[1-a_m^R a_f^R s_f^R -a_m^W a_f^R (1-s_f^R)]$
**W**♀+R♂+W♂	$na_m^W a_f^W s_f^W$ （**W**♀+ W♂）	$na_m^R a_f^W (1-s_f^W)$ （**W**♀+ R♂）	$n[1-a_m^R a_f^W (1-s_f^W)- a_m^W a_f^W s_f^W]$
